# Editorial: Awake surgery for brain tumors and brain connectomics

**DOI:** 10.3389/fonc.2022.1094818

**Published:** 2022-12-23

**Authors:** Nicola Montemurro, Gianluca Trevisi

**Affiliations:** ^1^ Department of Neurosurgery, Azienda Ospedaliera Universitaria Pisana (AOUP), University of Pisa, Pisa, Italy; ^2^ Department of Neurosciences, G d’Annunzio University Chieti-Pescara, Imaging and Clinical Sciences, Italy

**Keywords:** neurosurgery, awake surgery, brain tumor, brain connectomics, connectome, white matter dissection

Awake craniotomy is a valuable surgical approach to identify and preserve functional areas of the brain during removal of primary brain tumors, which can be performed safely with extremely low complications and failure rates, regardless of ASA classification, smoking status, frequency of seizures and location and size of tumor (1). The identification of functional area, especially in adult low-grade glioma, led to the improvement of the long-term quality of life of patients (2–4). This emerging surgical approach that allows to map the brain’s connections led to a change in thinking from a modular narrative of brain organization to a meta-network perspective. The risk-benefit ratio of surgery improves thanks to this greater comprehension of the connectome, enhancing intraoperative awake surgery and mapping and monitoring the so-called non-speaking areas, (2) enhancing preoperative information, enabling an ideal selection of surgical tasks in accordance with the patient’s wishes, (3) developing oncological resection surgery with disconnection, and (4) defining an individual multiphase surgical strategy will enable surgical resection in those “eloquent” areas in accordance with a localizationist dogma (5).

The purpose of this special issue is to analyze new research approaches to lower morbidity, analyze the effects of emerging technology and epidemiology on primary brain tumors, highlight the value of awake craniotomies during surgery, and highlight the significance of brain connectomics during white matter dissection. In a systematic review of surgical indication and glioma patients’ eligibility for awake surgery, Fiore et al. reported 45 studies with a total of 2645 glioblastoma patients. Psychological illnesses and anxiety are frequently cited as factors that rule someone out of awake surgery. Unwillingness brought on by psychological issues and anxiety has frequently been mentioned as an exclusion factor for awake surgery.

A meta-network perspective on neural processing that stresses the crucial role of dynamic connections across functional circuits in enabling sophisticated and flexible goal-directed behavior is the result of advancements in brain connectome research. Continuous multitasking in time-limited awake patients during intraoperative mapping is advised, according to Duffau et al., because it reflects desynchrony and synchrony within and between neural networks and improves the sensitivity of behavioral monitoring by improving cognitive functions. They also demonstrated the practical implications of this new intraoperative mapping paradigm in preserving whole-network dynamics during low-grade glioma surgery, showing that this seems to be a necessary step in preserving whole-network dynamics. Axelson et al. investigated whether continuous subcortical monopolar short-circuit stimulation (STS) with a suction probe is also helpful for cortical language mapping and found that STS-induced language disturbances at low intensities (≤ 5 mA) produced, in the same subcortical areas, language errors similar to bidirectional Penfield stimulation (PS), while STS language impairments at ≥ 7.5 mA or no effect of stimulation were usually associated with a negative PS, which seemed to indicate a safe distance to language areas. The only previous study (6) comparing STS with conventional PS found no significant differences in the number of electrical stimulation-induced mistakes (articulation, anomia, and paraphasia) between the two methods. In awake surgery, where neuroimaging might be imprecise and patient’s progressive fatigue affects basic language skills and test cooperation, this technique can be a time-saving technique that is especially crucial. Intraoperative testing must be customized by neurosurgeons and their teams to the patient’s demands, medical history, and tumor’s location and features. Based on a better comprehension of the ongoing interaction between neural circuits and the course of glioma, a better prediction of the neuroplasticity reserve for each patient at any given time would optimize multiphase and multimodal individual management of the disease prognosis and of the next treatments prior to the onset of functional decline (2, 7).

Finally, interesting findings have been reported from studies of local anesthetics and benzodiazepines. Together, the neurosurgeon and neuroanesthesiologist will decide which anesthetic is best for each patient. Patients must be conscious throughout surgery, and it is essential to provide painkillers and local anesthetics to the scalp lobe, the surgical site, and the temporalis muscle to guarantee a safe procedure (8). During an active craniotomy, it is essential to employ local anesthetics such ropivacaine effectively. But as described by Sato et al., who used a total dose of up to 5.0 mg/kg in an awake craniotomy with repeated anesthetic administration, resulting in a safe procedure, significant doses of ropivacaine can be administered safely during awake craniotomy. Similarly, the use of benzodiazepines for sedation in some patients with brain tumors has been linked to the recurrence of neurological impairments, including ataxia, motor weakness, and limb incoordination, which can cause complications for the patient as well as issues with the procedure. Its use in sedation should therefore be carefully considered by multidisciplinary care teams, especially in patients with preoperative neurologic impairments. The use of midazolam during awake craniotomies was associated with postoperative neurological deficits, according to a retrospective review by Plitman et al., which continues to support the theory that the increase in neurological deficits following benzodiazepine sedation may be caused by the unmasking of preoperative neurological deficits.

In conclusion, the Editors thank all the authors, reviewers and members of the editorial board for taking their time to participate and contribute to this Research Topic. We hope that this Research Topic can inspire future and new research approaches in the field of awake surgery and brain connectomics.

## Author contributions

All authors listed have made a substantial, direct, and intellectual contribution to the work and approved it for publication.
